# Mutations in *NFKB2* and potential genetic heterogeneity in patients with DAVID syndrome, having variable endocrine and immune deficiencies

**DOI:** 10.1186/s12881-014-0139-9

**Published:** 2014-12-19

**Authors:** Thierry Brue, Marie-Hélène Quentien, Konstantin Khetchoumian, Marco Bensa, José-Mario Capo-Chichi, Brigitte Delemer, Aurelio Balsalobre, Christina Nassif, Dimitris T Papadimitriou, Anne Pagnier, Caroline Hasselmann, Lysanne Patry, Jeremy Schwartzentruber, Pierre-François Souchon, Shinobu Takayasu, Alain Enjalbert, Guy Van Vliet, Jacek Majewski, Jacques Drouin, Mark E Samuels

**Affiliations:** Aix-Marseille University, Centre de Recherche en Neurobiologie et Neurophysiologie de Marseille (CRN2M), Centre National de la Recherche Scientifique, Unité Mixte de Recherche 7286, Faculté de Médecine de Marseille, 13344 Marseille, France; Assistance Publique-Hôpitaux de Marseille (APHM), Department of Endocrinology, Centre de Référence des Maladies Rares d’Origine Hypophysaire, Hôpital de la Timone, 13005 Marseille, France; Laboratoire de Génétique moléculaire, Institut de recherches cliniques de Montréal, 110 Avenue des Pins Ouest, Montréal, QC H2W 1R7 Canada; Ospedale Bufalini, Department of Paediatrics, Cesena, FC Italy; Centre de Recherche du CHU Ste-Justine, 3175 Cote Ste-Catherine, Montreal, QC Canada; Departments of Endocrinology and of Pediatrics, Centre Hospitalier Robert Debré, 51092 Reims, France; Department of Pediatric-Adolescent Endocrinology and Diabetes, Athens Medical Center, Athens, Greece; Clinique universitaire de pédiatrie, CHU de Grenoble, Grenoble, France; Department of Human Genetics, McGill University, Montreal, QC Canada; Endocrinology Service and Research Center, Department of Pediatrics, CHU Ste-Justine, University of Montreal, Montreal, QC Canada; Department of Medicine, University of Montreal, Montreal, QC Canada

## Abstract

**Background:**

DAVID syndrome is a rare condition combining anterior pituitary hormone deficiency with common variable immunodeficiency. *NFKB2* mutations have recently been identified in patients with ACTH and variable immunodeficiency. A similar mutation was previously found in *Nfkb2* in the immunodeficient *Lym1* mouse strain, but the effect of the mutation on endocrine function was not evaluated.

**Methods:**

We ascertained six unrelated DAVID syndrome families. We performed whole exome and traditional Sanger sequencing to search for causal genes. *Lym1* mice were examined for endocrine developmental anomalies.

**Results:**

Mutations in the *NFKB2* gene were identified in three of our families through whole exome sequencing, and in a fourth by direct Sanger sequencing. *De novo* origin of the mutations could be demonstrated in three of the families. All mutations lie near the C-terminus of the protein-coding region, near signals required for processing of NFΚB2 protein by the alternative pathway. Two of the probands had anatomical pituitary anomalies, and one had growth and thyroid hormone as well as ACTH deficiency; these findings have not been previously reported. Two children of one of the probands carried the mutation and have to date exhibited only an immune phenotype. No mutations were found near the C-terminus of *NFKB2* in the remaining two probands; whole exome sequencing has been performed for one of these. *Lym1* mice, carrying a similar *Nfkb2* C-terminal mutation, showed normal pituitary anatomy and expression of proopiomelanocortin (*POMC*).

**Conclusions:**

We confirm previous findings that mutations near the C-terminus of *NFKB2* cause combined endocrine and immunodeficiencies. *De novo* status of the mutations was confirmed in all cases for which both parents were available. The mutations are consistent with a dominant gain-of-function effect, generating an unprocessed *NFKB2* super-repressor protein. We expand the potential phenotype of such *NFKB2* mutations to include additional pituitary hormone deficiencies as well as anatomical pituitary anomalies. The lack of an observable endocrine phenotype in *Lym1* mice suggests that the endocrine component of DAVID syndrome is either not due to a direct role of NFKB pathways on pituitary development, or else that human and mouse pituitary development differ in its requirements for NFKB pathway function.

**Electronic supplementary material:**

The online version of this article (doi:10.1186/s12881-014-0139-9) contains supplementary material, which is available to authorized users.

## Background

Deficit in anterior pituitary function (hypopituitarism) and common variable immune deficiency (CVID, MIM [607594]) represent two very different clinical presentations. Mutations in several genes have been associated with each of these conditions, but until recently no gene has been associated with deficiencies in both systems in the same patients. Several years ago we identified such patients in three families as part of a national screen for hypopituitarism in France, and defined a novel disorder which we named DAVID syndrome (for Deficient Anterior pituitary with Variable Immune Deficiency) [1]. At the time it was not obvious whether these patients represented a shared molecular etiology or a coincidental overlap of two otherwise uncommon pediatric conditions, although there were at least two previous isolated case reports with similar clinical descriptions [2,3]. Chen *et al* recently reported mutations in the *NFKB2* gene in two families from Utah with features consistent with our definition of DAVID syndrome (immunodeficiency with hypogammaglobulinemia, plus central pituitary deficiency), one mutation of which was consistent with *de novo* germ line origin [4]. Several additional groups have since reported mutations near the C-terminus of *NFKB2*, in patients with features overlapping DAVID syndrome, although not all patients showed the endocrine aspect of the phenotype [5-7].

## Methods

Research ethics approval was obtained from the review boards of the Centre de Recherche du CHU Ste-Justine, and of the Assistance Publique-Hôpitaux de Marseille. Written informed consent for the study and for publication of the patients’ anonymous details and images were obtained from the participants or their parents. Clinical studies were performed as described in the Additional file 1. All experimental procedures with laboratory animals were approved by the IRCM Animal Protection Committee and followed guidelines and regulations of the Canadian Council of Animal Care. Whole exome sequencing on DNA from peripheral blood leukocytes was performed using standard methods and as previously described (see Additional file 1 for additional details) [8]. PCR-based Sanger sequencing with fluorescent capillary electrophoresis was performed using standard methods, and results were visualized using Mutation Surveyor (Soft Genetics, Inc.) Numbering in this study refers to Refseq entry NM_001077494.3, equivalent to isoform a, consistent with the usual amino acid numbering in functional studies of NFKB2 protein and the non-canonical processing pathway. Nucleotide numbering for mutations begins at the first A of the initiating methionine ATG.

*Lym1* mice were obtained from the Walter and Eliza Hall Institute (Victoria, Australia). Histology and immunohistochemistry were performed as previously described [9]. See Additional file 1 for additional details.

## Results and discussion

**Figure 1:**
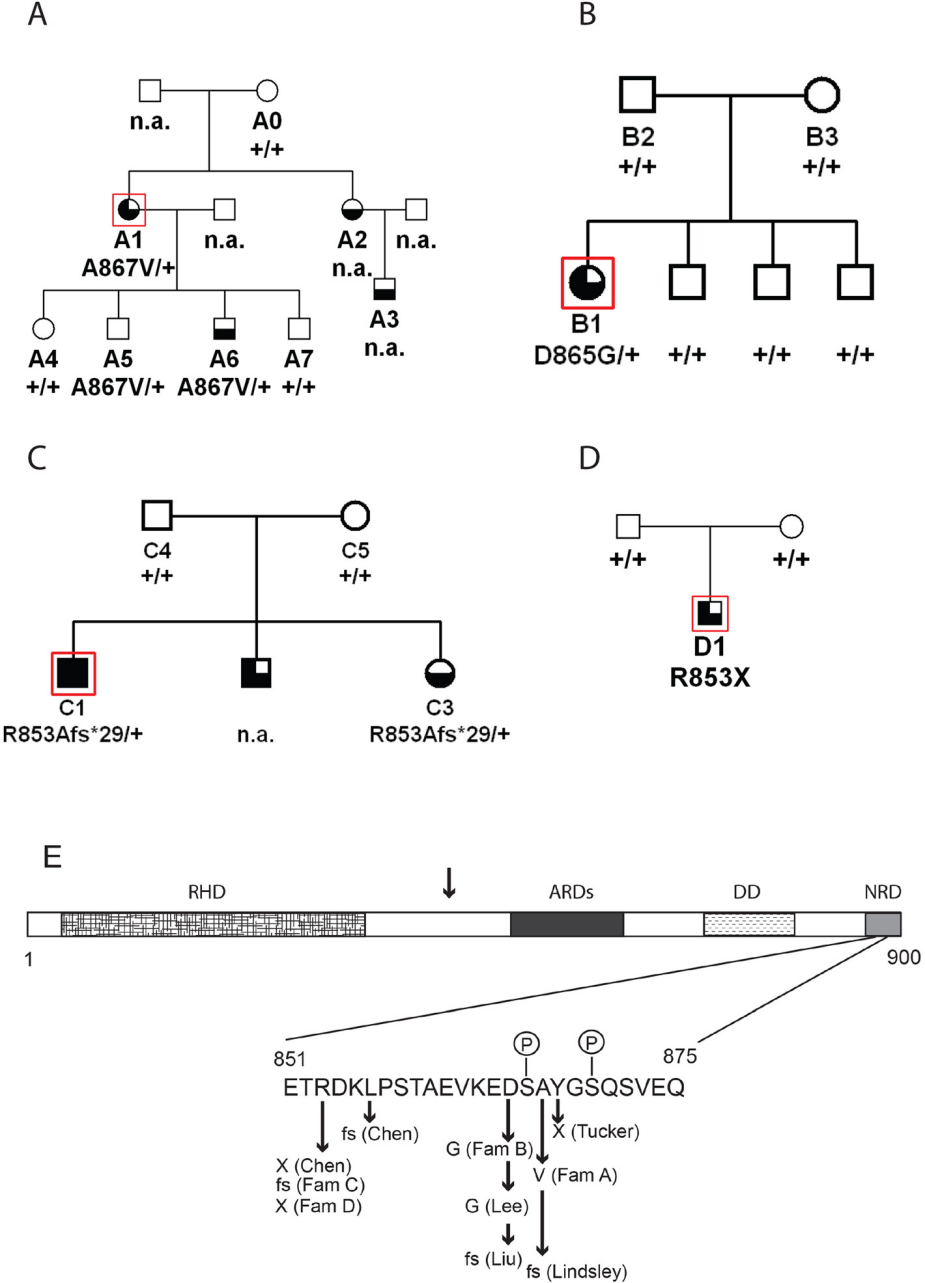
**DAVID syndrome families and** ***NFKB2*** **gene structure with mutations. A-D**, pedigrees showing genotypes of sequenced individuals. Symbols: Filled, immunodeficiency, ACTH deficiency, GH deficiency; lower half filled, immunodeficiency; lower half plus upper left quadrant filled, immunodeficiency, ACTH deficiency; open, unaffected or unknown. n.a. DNA and clinical information not available. +/+, no mutation at any of the three sites found in the families. **E**, structure of the NFκB2 protein (isoform A numbering), indicating the major functional domains, regulated phosphorylated serines 866 and 870, and sites of mutations reported here, by Chen, Liu, Lee, Lindsley *et al*, or at the orthologous site in the *Lym*1 mouse (Tucker *et al*). The vertical arrow above the domain cartoon indicates approximate site of proteolytic cleavage of p100, which releases the amino-terminal p52 active fragment. RHD, rel homology domain; ARDs, ankyrin repeat domains; DD, death domain; NRD, NIK response domain.

Our original French probands, recruited from different geographic regions through the “Genhypopit” network, [10] were noted to have immunodeficiencies of varying severity with recurrent childhood infections and hypogammaglobulinemia, in addition to ACTH deficiency (patients A1, B1, C1, C2, see Figure 1, our original publication for full clinical details, and Additional file 1 for updated phenotypes; C2 was deceased prior to this study) [1]. Patients A2, A3 and C3 had immune but not endocrine deficiencies. Patient C1 also had a growth hormone deficiency, and this patient has since developed central (*i.e.* not thyroidal) hypothyroidism. The endocrine deficiencies appear to be longstanding, not acute secondary to current infections. MRI scans of the pituitary were performed for the probands, with hypoplasia observed for patient B1 but not A1 or C1 (Additional file 1: Figure S1A, B, C1, 2). Since the original report, two new carriers were ascertained in family A, A5 and A6 (see below and Additional file 1: Table S1 for clinical details).

We performed whole exome sequencing on DNA from A1, B1 and C1. In the exome data, only one gene carried a rare, protein-altering variant in all three probands, the gene *NFKB2* (MIM [164012]). In *NFKB2*, proband A1 was heterozygous for missense mutation p.A867V, proband B1 was heterozygous for missense mutation p.D865G, and proband C1 was heterozygous for an 8 bp frameshift deletion, p.R853Afs*29 (Figure 1A, B, C). All three mutations were verified by PCR-based Sanger fluorescent sequencing (Additional file 1: Figure S3A-E). Sequencing confirmed that both parents were negative in peripheral blood DNA for the mutations found in the probands in families B and C, as was the one sampled parent in family A. In families A and B, all of the sampled unaffected relatives were negative for the familial mutation (data not shown). Two children of A1, A5 and A6, were heterozygous for the familial mutation (data not shown). One child (A6) showed a clinical phenotype with recurrent infections and immunodeficiency, while A5 had moderate immunoglobulin deficiency with no clinical signs as yet. In family C, affected sibling C3 of the proband also carried the familial mutation. No additional pathogenic variants were found in any of the probands in targeted exons of genes otherwise known to cause hypopituitarism (see Additional file 1). There were rare protein-altering variants detected in several other genes associated with common variable or severe immunodeficiency, some in B1 and some in C1, but none in any two probands together (see Additional file 1: Table S2).

A fourth patient with DAVID syndrome was independently ascertained, previously only reported in abstract form [11]. This patient (D1, see Figure 1, Additional file 1: S2)) was asthenic with immunodeficiency from an early age, suffered hair loss and onychodystrophy, and had low ACTH before and after CRH stimulation. All other pituitary functions were normal, and MRI showed hypoplastic anterior pituitary (Additional file 1: Figure S1D, see Additional file 1 for more details). The two C-terminal coding exons of *NFKB2* were directly sequenced in DNA from patient D1, using PCR-based Sanger technology. The patient was heterozygous for a truncating mutation also at amino acid position 853, p.R253X (Figure 1D). This was not the same as the frameshift mutation at the same residue in family C. The mutation was not present in blood DNA from either parent (data not shown).

All variants have been submitted to ClinVar and assigned preliminary accession numbers pending database curation: c.2556_2563del SCV000172085; c.2594A > G SCV000172086; c.2600C > T SCV000172087; c.2557C > T SCV000172088.

Our genetic findings strongly implicate *NFKB2* as the causal gene for DAVID syndrome in our patients, particularly taking into account similar mutations found by two other groups in similar patients. In all we identified four different heterozygous mutations, two protein-truncating and two missense variants, near the C-terminus of the protein coding region of *NFKB2*, a region required for the correct processing of the primary translation product (Figure 1E) [12,13]. In the three families (B, C, D) where both parents could be sampled, neither parent carried the mutation in DNA from peripheral lymphocytes, consistent with the mutations being germline *de novo*; family A with only one parent available is also compatible with this model of transmission. The occurrence of two affected siblings in family C is completely consistent with the proposed *de novo* germ line origin of the mutations. Although uncommon, such mutations may arise in mitotic germ line clones, either in testes or ovaries, and clonally expand, giving rise to mutation-carrying post-meiotic gametes repeatedly, potentially over a period of years. Intrafamilial recurrence in multiple affected siblings with presumptive *de novo* pre-meiotic germ line origin has been reported in numerous studies of unrelated genetic disorders [14-18].

The mutation in family B of Chen *et al* is identical to the mutation in our family D at the nucleotide and protein level (p.R253X, Figure 1E) [4]. These probably represent independent mutational events, although the ethnicity of their family was not reported. In the family reported by Liu *et al.*, the phenotype co-segregates with a frameshift mutation beginning one amino acid upstream of phosphorylated serine 865. It should be noted that hypoplastic pituitary anatomy as visualized by MRI was not reported by either Chen or Liu *et al*, whereas two of our patients have anatomical pituitary defects. Similarly, the mutation p.D865G reported by Lee *et al* is the same as that found in our family B [5]. Lee *et al* demonstrated that this mutation deleteriously affected NFKB2 protein processing in response to activation of the non-canonical pathway. Oddly, all three patients of Lee *et al* are reported to have alopecia areata, which we did not observe in our patient B1, whereas their patients lacked an overt endocrine deficiency.

**Figure 2:**
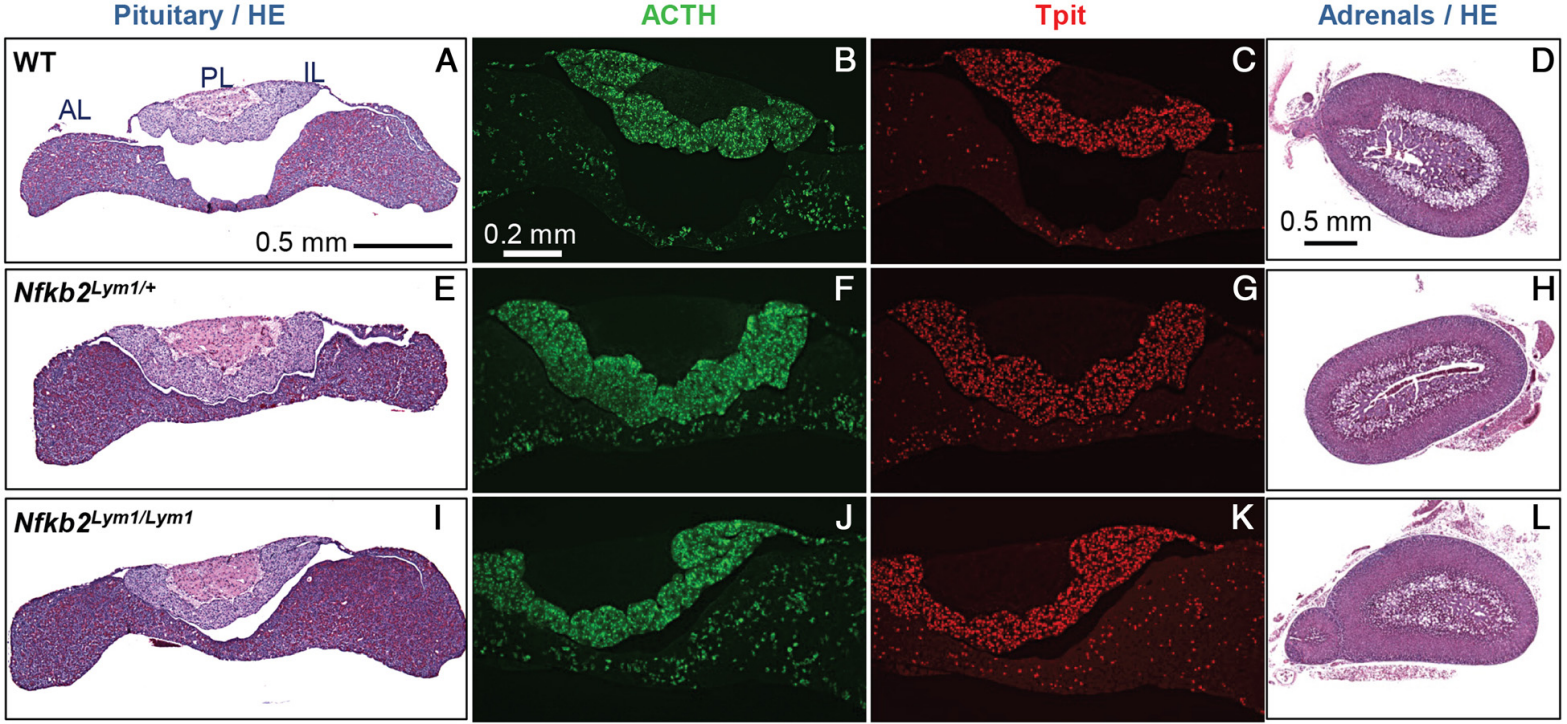
**Histology of** ***Lym1*** **mutant mouse pituitaries and adrenals.** HE staining of pituitaries **(A, E, I)** and adrenals **(D, H, L)** from WT **(A, D)**, heterozygous (*Nfkb2* ^*Lym1/+*^) or homozygous (*Nfkb2* ^*Lym1/Lym1*^) newborn mice. Staining of pituitaries with anti-ACTH **(B, F, J)** or anti-Tpit **(C, G, K)** antibodies in wild type, heterozygous, and homozygous mutant mice respectively. AL: anterior lobe, IL: intermediate lobe, PL: posterior lobe. Scale bars are shown.

The unique association of ACTH deficiency with combined variable immunodeficiency in DAVID syndrome suggested that *NFKB2* might have a critical role in pituitary development, particularly for differentiation of ACTH-producing corticotrope cells. A similar mutation near the C-terminus of the murine *Nfkb2* ortholog was previously identified in a large scale mouse mutagenesis screen for cellular immunodeficiency [19]. This *Lym1* strain shows severely reduced viability as homozygotes, although heterozygotes are viable and fertile. The mice show multiple aspects of immunodeficiency similar to DAVID syndrome, including a reduced B cell compartment and reduced antigen response with hypogammaglobulinemia and autoimmune response in heterozygotes. However the endocrine status of these mice was not previously described, nor was the anatomical development of their hypothalamus and pituitary. We assessed pituitary development in the *Lym1* mice at various ages. Unexpectedly, the gross morphology of pituitaries from wild-type and *Lym1*heterozygous or homozygous mutant pituitaries did not reveal any obvious anatomical defect either in young mice (Figure 2A, E, I), or in older animals (Additional file 1: Figure S4). Expression of *Pomc* was assessed using ACTH immunofluorescence, and the differentiation status of corticotropes and melanotropes was assessed using immunostaining for Tpit (Tbx19) Differentiation of anterior lobe corticotropes and intermediate lobe melanotropes appeared normal in distribution and cell numbers (Figure 2C, G, K, S4) and expression of *Pomc* was not obviously affected by the *Lym1* mutation (Figure 2B, F, G, S4). Histological analysis of adrenals from mice of the three genotypes also did not reveal any defect (Figure 2D, H, L). In separate experiments using expression array profiling, we have documented that both *Nfkb1* and *Nfkb2* are expressed in the adult mouse anterior pituitary (see Additional file 1: Figure S5).

NFκB2 is synthesized as a large precursor (p100) that must be processed to release the active N-terminal rel-homology domain (RHD, p52). Processing of NFκB2 is tightly regulated via the non-canonical pathway, with most protein remaining unprocessed until the cell receives an appropriate signal via receptors such as LTβR, BAFFR, RANK or CD40 [12,20,21]. Signalling leads to phosphorylation of specific serines near the C-terminus of NFκB2, including S866 and S870, via a pathway that employs the NIK and IKKA kinases [12]. This in turn leads to cleavage by the proteasome to produce the RHD p52 fragment. The occurrence of missense mutations bracketing S866 in two of our families suggests that these residues are important for recognition of the site by its kinase. Unprocessed full-length NFκB2 p100 can act as an IκB-like inhibitor [12,22]. Mutations in *NFKB2* leading to a protein that is unable to proceed via the non-canonical pathway, but is otherwise functional, are therefore anticipated to generate a dominant negative repressor, which could block the action of multiple RHD-dimers. The tight clustering of six different mutations in human DAVID syndrome patients in the region of *NFKB2* regulated by the non-canonical pathway (Figure 1E) is likely related to the specificity and rarity of the phenotype. It should be noted that mutations in *NFKB2* have not been reported in cases of isolated anterior pituitary deficiency, lacking an immunodeficiency component.

The pathology we observed in the *Lym1* mice is inconsistent with an effect of the *Nkfb2* mutation in that strain at the level of primary pituitary development. Alternatively, the human endocrine phenotype might potentially result from an autoimmune reaction, consistent with the observed alopecia in several DAVID patients. *Nfkb2* has been shown to play a role in acquired self-tolerance in knockout mice [23]. Mice accumulating p100 as a result of defects in or upstream of *Nfkb2* show mild osteopetrosis, [19,24] and the RHD system is known to play a role in bone metabolism [25]. However, our DAVID syndrome patients carrying *NFKB2* mutations have normal bone mineral density (T. Brue and M.-H. Quentien, *pers. comm*.).

The occurrence of GH and TSH deficiencies in our patient C1, as well as the abnormal pituitary anatomy observed in patients B1 and D1, constitute an expansion of the phenotype for this gene defect. We have previously reported that severe neonatal cortisol deficiency may be associated with transient GH deficiency [26] but an association with TSH deficiency is a novel observation. The subclinical phenotype of patient A5 is indicative of variable penetrance even in the same family, or a possibly delayed onset of the phenotype. The terminology of ‘central adrenal’ deficiency in other papers describing *NFKB2* mutations may be overly restrictive, as the endocrine deficiencies among our patients are not restricted to hormones affecting adrenal function. A corticotropin-releasing factor test of patient D1 was consistent with a pituitary and not an hypothalamic defect.

We recently ascertained a fifth case (E1) with DAVID syndrome, in an unrelated French patient with both parents unaffected (but not sampled). Sanger sequencing of the two C-terminal coding exons of *NFKB2* was negative for any variation. Thus the phenotype of this patient remains unexplained.

A sixth, French-Canadian patient (F1), was ascertained after being hospitalized at age 17 years for a renal granuloma with immunoglobulin M-complex glomerulonephritis, low IgG levels, and a diagnosis of possible common variable immunodeficiency [27]. She was referred to Endocrinology due to primary amenorrhea and was found to have hypothalamic hypogonadism and severe ACTH and growth hormone deficiencies, although she was not of short stature [28]. Pituitary MRI showed a small anterior pituitary and a thin pituitary stalk with an ectopic posterior pituitary, suggesting a congenital defect [29]. Exome sequencing of DNA from this patient showed no pathogenic variants in *NFKB2*, whereas all coding exons of the gene were well covered. As is typical, several hundred heterozygous rare potentially pathogenic variants were identified in the complete exome analysis of this patient. The results for patients E1 and F1 suggest that genetic heterogeneity is likely for DAVID syndrome, and that variable endocrine presentation may be a hallmark of the disorder.

The rel homology domain transcription factor signalling pathway is an active target of therapies for cancers especially breast and multiple myeloma [30,31]. Our results caution that broad inhibition of these pathways, or specific inhibition of NFκB2 processing, could have long-term deleterious effects on central endocrine function. Monitoring endocrine function may be indicated as a component of drug trials targeting NFκB pathways.

## Conclusions

We confirm previous findings that mutations near the C-terminus of *NFKB2* cause combined endocrine and immunodeficiencies. *De novo* status of the mutations was confirmed in all cases for which both parents were available. The mutations are consistent with a dominant gain-of-function effect, generating an unprocessed *NFKB2* super-repressor protein. We expand the potential phenotype of such *NFKB2* mutations to include additional pituitary hormone deficiencies as well as anatomical pituitary anomalies. The lack of an observable endocrine phenotype in *Lym1* mice suggests that the endocrine component of DAVID syndrome is either not due to a direct role of NFΚB pathways on pituitary development, or else that human and mouse pituitary development differ in its requirements for NFΚB pathway function.

## Additional file

Additional file 1:
**Clinical description of the patients.**

## References

[1] Quentien MH, Delemer B, Papadimitriou DT, Souchon PF, Jaussaud R, Pagnier A, Munzer M, Jullien N, Reynaud R, Galon-Faure N, Enjalbert A, Barlier A, Brue T (2012). Deficit in anterior pituitary function and variable immune deficiency (DAVID) in children presenting with adrenocorticotropin deficiency and severe infections. J Clin Endocrinol Metab.

[2] Younes JS, Secord EA (2002). Panhypopituitarism in a child with common variable immunodeficiency. Ann Allergy Asthma Immunol.

[3] Tovo PA, Lala R, Martino S, Pastorelli G, De Sanctis C (1991). Isolated adrenocorticotropic hormone deficiency associated with common variable immunodeficiency. Eur J Pediatr.

[4] Chen K, Coonrod EM, Kumanovics A, Franks ZF, Durtschi JD, Margraf RL, Wu W, Heikal NM, Augustine NH, Ridge PG, Hill HR, Jorde LB, Weyrich AS, Zimmerman GA, Gundlapalli AV, Bohnsack JF, Voelkerding KV (2013). Germline mutations in NFKB2 implicate the noncanonical NF-kappaB pathway in the pathogenesis of common variable immunodeficiency. Am J Hum Genet.

[5] Lee CE, Fulcher DA, Whittle B, Chand R, Fewings N, Field M, Andrews D, Goodnow CC, Cook MC (2014). Autosomal-dominant B-cell deficiency with alopecia due to a mutation in NFKB2 that results in nonprocessable p100. Blood.

[6] Lindsley AW, Qian Y, Valencia CA, Shah K, Zhang K, Assa'ad A (2014). Combined immune deficiency in a patient with a novel NFKB2 mutation. J Clin Immunol.

[7] Liu Y, Hanson S, Gurugama P, Jones A, Clark B, Ibrahim MA (2014). Novel NFKB2 mutation in early-onset CVID. J Clin Immunol.

[8] Majewski J, Schwartzentruber JA, Caqueret A, Patry L, Marcadier J, Fryns JP, Boycott KM, Ste-Marie LG, McKiernan FE, Marik I, Van Esch H, Michaud JL, Samuels ME, FORGE Canada Consortium (2011). Mutations in NOTCH2 in families with Hajdu-Cheney syndrome. Hum Mutat.

[9] Budry L, Balsalobre A, Gauthier Y, Khetchoumian K, L'Honore A, Vallette S, Brue T, Figarella-Branger D, Meij B, Drouin J (2012). The selector gene Pax7 dictates alternate pituitary cell fates through its pioneer action on chromatin remodeling. Genes Dev.

[10] Reynaud R, Gueydan M, Saveanu A, Vallette-Kasic S, Enjalbert A, Brue T, Barlier A (2006). Genetic screening of combined pituitary hormone deficiency: experience in 195 patients. J Clin Endocrinol Metab.

[11] Locatelli C, Lugaresi L, Zerial M, Bensa M, Pocecco M (2006). Isolated adrenocorticotropic hormone deficiency in a child with common variable immunodeficiency. European Society for Paediatric Endocrinology 45th Annual Meeting.

[12] Sun SC (2012). The noncanonical NF-kappaB pathway. Immunol Rev.

[13] Xiao G, Harhaj EW, Sun SC (2001). NF-kappaB-inducing kinase regulates the processing of NF-kappaB2 p100. Mol Cell.

[14] Gloyn AL, Cummings EA, Edghill EL, Harries LW, Scott R, Costa T, Temple IK, Hattersley AT, Ellard S (2004). Permanent neonatal diabetes due to paternal germline mosaicism for an activating mutation of the KCNJ11 Gene encoding the Kir6.2 subunit of the beta-cell potassium adenosine triphosphate channel. J Clin Endocrinol Metab.

[15] Stoppa-Vaucher S, Ayabe T, Paquette J, Patey N, Francoeur D, Vuissoz JM, Deladoey J, Samuels ME, Ogata T, Deal CL (2012). 46, XY gonadal dysgenesis: new SRY point mutation in two siblings with paternal germ line mosaicism. Clin Genet.

[16] Gauthier J, Champagne N, Lafrenière RG, Xiong L, Spiegelman D, Brustein E, Lapointe M, Peng H, Côté M, Noreau A, Hamdan FF, Addington AM, Rapoport JL, Delisi LE, Krebs MO, Joober R, Fathalli F, Mouaffak F, Haghighi AP, Néri C, Dubé MP, Samuels ME, Marineau C, Stone EA, Awadalla P, Barker PA, Carbonetto S, Drapeau P, Rouleau GA, S2D Team (2010). De novo mutations in the gene encoding the synaptic scaffolding protein SHANK3 in patients ascertained for schizophrenia. Proc Natl Acad Sci U S A.

[17] Kara-Mostefa A, Raoul O, Lyonnet S, Amiel J, Munnich A, Vekemans M, Magnier S, Ossareh B, Bonnefont JP (1999). Recurrent Williams-Beuren syndrome in a sibship suggestive of maternal germ-line mosaicism. Am J Hum Genet.

[18] Rantamaki T, Kaitila I, Syvanen AC, Lukka M, Peltonen L (1999). Recurrence of Marfan syndrome as a result of parental germ-line mosaicism for an FBN1 mutation. Am J Hum Genet.

[19] Tucker E, O'Donnell K, Fuchsberger M, Hilton AA, Metcalf D, Greig K, Sims NA, Quinn JM, Alexander WS, Hilton DJ, Kile BT, Tarlinton DM, Starr R (2007). A novel mutation in the Nfkb2 gene generates an NF-kappa B2 "super repressor". J Immunol.

[20] Mordmuller B, Krappmann D, Esen M, Wegener E, Scheidereit C (2003). Lymphotoxin and lipopolysaccharide induce NF-kappaB-p52 generation by a co-translational mechanism. EMBO Rep.

[21] Dejardin E (2006). The alternative NF-kappaB pathway from biochemistry to biology: pitfalls and promises for future drug development. Biochem Pharmacol.

[22] Scheinman RI, Beg AA, Baldwin AS (1993). NF-kappa B p100 (Lyt-10) is a component of H2TF1 and can function as an I kappa B-like molecule. Mol Cell Biol.

[23] Zhu M, Chin RK, Christiansen PA, Lo JC, Liu X, Ware C, Siebenlist U, Fu YX (2006). NF-kappaB2 is required for the establishment of central tolerance through an Aire-dependent pathway. J Clin Invest.

[24] Seo Y, Fukushima H, Maruyama T, Kuroishi KN, Osawa K, Nagano K, Aoki K, Weih F, Doi T, Zhang M, Ohya K, Katagiri T, Hosokawa R, Jimi E (2012). Accumulation of p100, a precursor of NF-kappaB2, enhances osteoblastic differentiation in vitro and bone formation in vivo in aly/aly mice. Mol Endocrinol.

[25] Boyce BF, Xing L, Franzoso G, Siebenlist U (1999). Required and nonessential functions of nuclear factor-kappa B in bone cells. Bone.

[26] McEachern R, Drouin J, Metherell L, Huot C, Van Vliet G, Deal C (2011). Severe cortisol deficiency associated with reversible growth hormone deficiency in two infants: what is the link?. J Clin Endocrinol Metab.

[27] Benoit G, Lapeyraque AL, Sartelet H, Saint-Cyr C, Le Deist F, Haddad E (2009). Renal granuloma and immunoglobulin M-complex glomerulonephritis: a case of common variable immunodeficiency?. Pediatr Nephrol.

[28] Hasselmann C, Samuels ME, Van Vliet G (2014). Goliath, a Variant of DAVID Syndrome?. 53rd Annual Meeting of the European Society of Pediatric Endocrinology.

[29] Deal C, Hasselmann C, Pfaffle RW, Zimmermann AG, Quigley CA, Child CJ, Shavrikova EP, Cutler GB, Blum WF (2013). Associations between pituitary imaging abnormalities and clinical and biochemical phenotypes in children with congenital growth hormone deficiency: data from an international observational study. Horm Res Paediatr.

[30] Xiao G, Rabson AB, Young W, Qing G, Qu Z (2006). Alternative pathways of NF-kappaB activation: a double-edged sword in health and disease. Cytokine Growth Factor Rev.

[31] Baud V, Karin M (2009). Is NF-kappaB a good target for cancer therapy? Hopes and pitfalls. Nat Rev Drug Discov.

