# Supplementary material for: Mutations in NFKB2 and potential genetic heterogeneity in patients with DAVID syndrome, having variable endocrine and immune deficiencies
Source: BMC Med Genet. 2014 Dec 19;15:139. doi: 10.1186/s12881-014-0139-9 (PMC4411703; doi:10.1186/s12881-014-0139-9)

Supplemental Materials for "Mutations in *NFKB2* and potential genetic heterogeneity in patients with DAVID syndrome, having variable endocrine and immune deficiencies"

Clinical description of the patients

We describe 4 unrelated families presenting with DAVID syndrome; i.e. combined variable immunodeficiency (CVID) associated with anterior pituitary hormone deficits especially ACTH deficiency (Fig.S1). Here, we provide an update of the phenotypes of previously reported patients, plus clinical descriptions of newly identified affected or unaffected members of these families.[1] In pedigree A, the index case is a girl (patient A1) diagnosed with isolated moderate immunoglobulin deficiency at age 8 y. At the age of 15 y, severe ACTH deficiency was diagnosed. A1 showed normal pituitary on MRI (Fig. S1A). Her sister (patient A2) and the only child of this patient (A3) were found to present with CVID without any overt endocrine phenotype. Unfortunately, DNA of A2 and A3 were not available for analysis. Patient A1 had 5 pregnancies with one spontaneous abortion and 4 live births in 7 years. Recently, her children (1 girl: A4 and 3 boys: A5-A7) were also studied and one of them (A6) had immune deficiency diagnosed as part of a familial screening, without any endocrine deficit (Table S1). During a period of discontinuation of immunoglobulin treatment for about a year A6 had repeat ear nose or throat infections despite antibacterial treatment. A6 is currently 9 years of age. A5 is currently 12 years of age and presents with moderate immunoglobulin deficit. He has no immunoglobulin treatment and has not experienced unusually severe infections. All other siblings had normal immune and endocrine parameters.

In pedigree B, the proband is a girl (B1) presenting with CVID and ACTH deficiency as previously described. An MRI scan showed pituitary hypoplasia (Fig. S1B). She was treated with immunoglobulins and cortisol replacement. As already reported for her parents and one of her brothers, her other two brothers were also recently found to have normal endocrine and immune parameters.

In pedigree C, a boy (C1) was diagnosed with variable immunodeficiency at age 4 y and treated with immunoglobulins. At age 9 y, cortisol and ACTH were found undetectable even after CRH test. He also had a partial GH deficiency. After initiation of cortisol and GH replacement therapies, he grew normally and severe infections stopped. Since our first report, the boy was examined at 15.2 y, and found to have a slightly decreased free T4 (10 pmol/l) and with

unexpectedly normal TSH. L-thyroxine was started. He grew and completed puberty normally. He recently had infections resulting in an increase of cortisol treatment. His growth and puberty were almost completed at 17 years of age. IGF1 was still low (169 ng/ml), indicating persistent partial GH deficiency. MRI scan showed a normal pituitary (Fig. S2C1, C2). A sister (patient C3) had normal pituitary function and a milder immunoglobulin deficit. Both parents had unremarkable phenotypes.

Patients in families A, B, and C had no significant ectodermal phenotypes, although A1 has reportedly reduced body hair.

Patient D1, previously reported only in brief, is a boy who was very asthenic with no symptoms of infections but with decreased immunoglobulins at 18 months (Table S1).[2] Immunoglobulin therapy was then started. At 4 years, he had rapidly expanding hair loss to the whole scalp, eyebrows and eyelashes associated with onychodystrophy of hands and feet (see Fig. S2). At the age of 7 years, he had major asthenia, chills, excessive sweating, pallor, prostration, tachycardia, mild psychomotor slowdown and cold extremities that revealed severe hypoglycemia (27mg/dl) and required intravenous glucose infusion. Hyperinsulinemia was excluded and fasting hypoglycemia was suspected. However, due to the severity and persistence of hypoglycemia,cortisol was measured and found very low (0.5 mg/dl) before and after an ACTH stimulation test. During another hypoglycemia episode (42 mg/dl), extremely low cortisol and ACTH were evidenced and there was no ACTH increase after CRH stimulation. All other pituitary functions were normal. MRI showed an hypoplastic anterior pituitary (Fig. S1D). After initiation of hydrocortisone replacement therapy, no further episodes of hypoglycemia occurred. At 12 years and 9 months of age, the boy was well and his puberty started. Levels of gonadotrophins and testosterone were in accordance with the developmental stage (FSH 1.8 UI/L; LH 3.5 UI/L and testosterone 17.2 nmol/L). At 15 years and 9 months, he had normal levels of gonadotrophins and testosterone (FSH 0.9 UI/L, LH 3,3 UI/L, testosterone 26.96 nmol/L) despite absent pubic and axillary hair. IGF-1 was normal (288 ng/mL). Thyroid ultrasonography revealed a gland of normal size with no structural anomalies. The parents of this boy are not consanguineous and both of them had unremarkable phenotype (apparently normal pituitary function and no recurrent or severe infections).

Sequencing

For exome sequencing array-capture we employed the Agilent SureSelect 50Mb oligonucleotide library for Illumina HiSeq chemistry.[3] After aligning reads to the human genome using an

implementation of the Burroughs-Wheeler algorithm (BWA), mean and median read depths of bases in consensus coding sequence exons were greater than 100-fold. Each sample had at least 10-fold coverage for 90% or more of coding exon bases. We quality-filtered initial variant calls using SAMtools, requiring at least 3 and >20% variant reads. Variants were annotated with respect to protein-coding potential using ANNOVAR. Common variants were filtered using data from the 1000 Genomes project, and various technical artifacts were controlled for by filtering with a set of in-house exomes of unrelated projects. Alignments were visualized using either the Integrative Genomics Viewer IGV (Broad Institute),[4, 5] or NextGene (Soft Genetics, Inc.)

Consistent with previous Sanger sequencing, no rare pathogenic variants were observed in the patient exome data for the genes *TBX19*, *LIF*, *IKZF1* or *IKZF4*, nor in other relevant endocrine system developmental genes: *GLI2, HESX1, LHX3, LHX4, MC2R, MRAP, OTX2, PITX2, POMC, POU1F1, SHH, SHOX, SOX2, or SOX3*.[6] The coding variants listed in Table 1 were observed in the exome data for 5 genes associated with or playing a known role in common variable or more severe immunodeficiency: *PRKCD, TRAF2, POLE, TNFRSF13B, IL2RG.*

No candidate pathogenic variants were observed in the proband exomes in the following additional immunodeficiency genes: *ICOS, CD19, TNFRSF13C, CD20, CD21, CD40, CD3E, RAG1, RAG2, SP110, DNMT3B* (Table S2).

Mouse studies

Lym1 mice in Balb/c background[7] were obtained from Dr Ben Kile at the Walter and Eliza Hall Institute of Medical Research (Victoria, Australia). Histology and immunohistofluorescence were performed on 4% paraformaldedyde-fixed, paraffin-embedded tissue sections as described.[8] Antibodies were used at the following dilutions: rabbit anti-Tpit (generated in our laboratory): 1:100; mouse anti-ACTH (Fitzgerald): 1:100. For Tpit antibodies, slides were incubated with a biotinylated anti-rabbit antibody (Vector) and revealed with Alexa Fluor 546-coupled streptavidin (Molecular Probes). ACTH detection was performed using Alexa Fluor 488-coupled anti-mouse antibody (Molecular Probes).

For genotyping to determine zygosity of mice, genomic DNA was prepared from tail biopsies as previously described.[9] The *Lym1* mutation corresponds to a T to A transversion, changing Tyrosine867 (TAT) to a termination codon (TAA). The following primers were used in high stringency PCR (annealing at 65°C) to discriminate WT and *Lym1* alleles:

WT allele : 257 bp
forward: G GTG AAA GAA GAC AGT GCC TAT
reverse: GTTGCTGTACCGTAAGTTGG

Lym1 allele : 257 bp
forward: G GTG AAA GAA GAC AGT GCC TAA
reverse: GTTGCTGTACCGTAAGTTGG

Array profiling of gene expression was performed as described, with isolated mouse pituitary neuroendocrine cells.

References

1. Quentien MH, Delemer B, Papadimitriou DT, Souchon PF, Jaussaud R, Pagnier A, Munzer M, Jullien N, Reynaud R, Galon-Faure N *et al*: **Deficit in anterior pituitary function and variable immune deficiency (DAVID) in children presenting with adrenocorticotropin deficiency and severe infections**. *The Journal of clinical endocrinology and metabolism* 2012, **97**(1):E121-128.
2. Locatelli C, Lugaresi L, Zerial M, Bensa M, Pococco M: **Isolated adrenocorticotropic hormone deficiency in a child with common variable immunodeficiency**. In: *European Society for Paediatric Endocrinology 45th Annual Meeting.* vol. 65. Rotterdam: Hormone Research in Pediatrics; 2006: 36.
3. Majewski J, Schwartzentruber JA, Caqueret A, Patry L, Marcadier J, Fryns JP, Boycott KM, Ste-Marie LG, McKiernan FE, Marik I *et al*: **Mutations in NOTCH2 in families with Hajdu-Cheney syndrome**. *Human mutation* 2011, **32**(10):1114-1117.
4. Robinson JT, Thorvaldsdottir H, Winckler W, Guttman M, Lander ES, Getz G, Mesirov JP: **Integrative genomics viewer**. *Nature biotechnology* 2011, **29**(1):24-26.
5. Thorvaldsdottir H, Robinson JT, Mesirov JP: **Integrative Genomics Viewer (IGV): high-performance genomics data visualization and exploration**. *Briefings in bioinformatics* 2013, **14**(2):178-192.
6. Samuels ME, Hasselmann C, Deal CL, Deladoey J, Van Vliet G: **Whole-exome sequencing: opportunities in pediatric endocrinology**. *Personalized Medicine* 2014, **11**(1):63-78.
7. Tucker E, O'Donnell K, Fuchsberger M, Hilton AA, Metcalf D, Greig K, Sims NA, Quinn JM, Alexander WS, Hilton DJ *et al*: **A novel mutation in the Nfkb2 gene generates an NF-kappa B2 "super repressor"**. *J Immunol* 2007, **179**(11):7514-7522.
8. Budry L, Balsalobre A, Gauthier Y, Khetchoumian K, L'Honore A, Vallette S, Brue T, Figarella-Branger D, Meij B, Drouin J: **The selector gene Pax7 dictates alternate pituitary cell fates through its pioneer action on chromatin remodeling**. *Genes & development* 2012, **26**(20):2299-2310.
9. Pulichino AM, Vallette-Kasic S, Tsai JP, Couture C, Gauthier Y, Drouin J: **Tpit determines alternate fates during pituitary cell differentiation**. *Genes & development* 2003, **17**(6):738-747.

Supplemental Tables

Table S1.

Patients	A5 (12y)	A6 (6 yr)	D1 (15 yr)
**Immunological values**			
IgG g/l	6.7 (8.5-13.0)	4.6* (8.1-10)	5.98(5.49-15.84)
IgG1	5.31 (4.0-11.0)	decreased*	
IgG2	1.33 (0.8-4.8)		
IgG3	0.18 (0.15-0.74)	decreased*	
IgG4	0.03 (0.01-1.58)		
IgA g/L	0.47 (0.75-2.60)	0.23* (1.2-6)	0.09 (0.61-3.48)
IgM g/L	0.39 (0.7-1.80)	0.21* (0.6-1.6)	<0.05 (0.23-2.59)
**Hormonal values**			
base line ACTH ng/L			undetectable
post CRF ACTH ng/L			undetectable
IGF1 ng/mL			288 (226-903)
peak GH mUI/l			
T4 ng/L			8.4 (9.3-16)
TSH mUI/L			5.290 (0.200-5.800)
PRL ug/L			12.2 (<24)
FSH UI/L			0.9 (1.5-12.4)
LH UI/L			3.3 (1.7-8.6)

* Ig substitution stopped during one year; immune investigations performed before restart of the treatment

Table S2.

Gene	Chrom.	Variant	Proband	Zygosity	Reported SNP	m.a.f.	Comments
PRKCD	3	p.300R>W	C1	Het	None	n.a.	A similar SNP, p.R300Q SNP has m.a.f. 0.05%, residue not conserved in lower vertebrates
TRAF2	9	p. 147E>D	B1	Het	rs199667825	n.a.	Residue well conserved, variant conservative
POLE	12	p. 259R>H	C1	Het	rs61732929	~1%	Residue well conserved
TNFRSF13B	17	p. 220V>A	C1	Het	rs56063729	1.5%	Residue not well conserved
IL2RG	X	p.348A>T	B1	Het	rs140216601	~0.03%	Residue not well conserved

Supplemental Figures

S1. MRI of pituitaries of DAVID syndrome patients. A) pituitary was of normal size, without visible lesion or sign of hypophysitis in patient A1 at 15 years; B) at about 5 years of age, patient B1 had pituitary hypoplasia; C) antehypophysis of patient C1 (9 years old) was normal; D) hypoplasic antehypophysis was observed in patient D1 at 7 years of age. White arrows show pituitary.

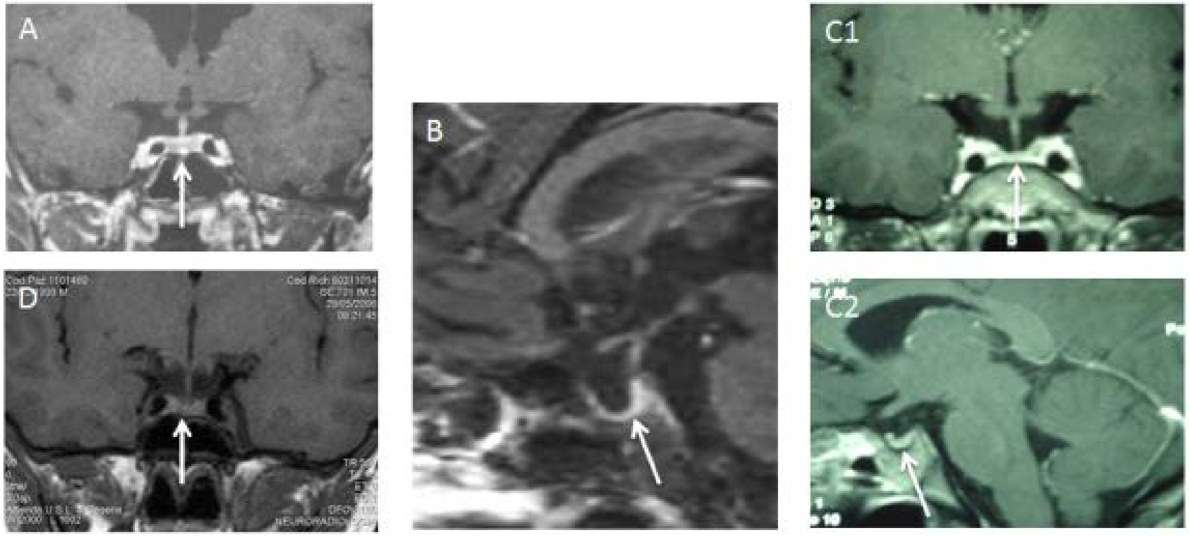

S2. Patient D1, documenting alopecia and onychodystrophy between 4 years of age (A) and 7 years of age (B) resulting in loss of hair, eyelashes and eyebrows. Picture (C) shows onychodystrophy with characteristics of ungual candidiasis (periungual inflammation, ungual thickness and onycholisis).

A

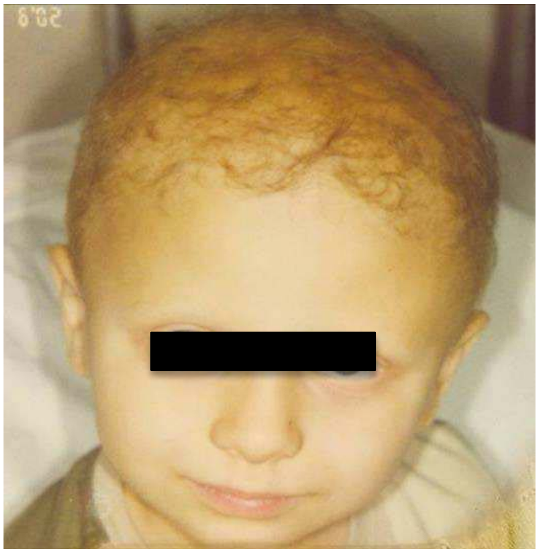

B

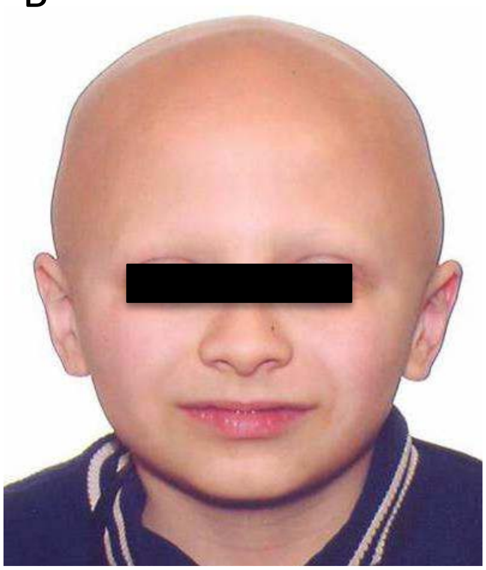

C

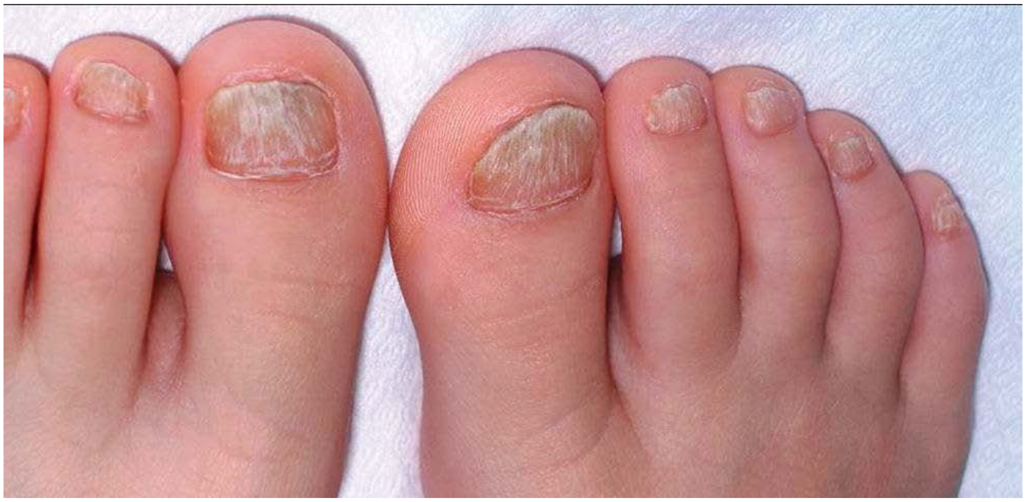

S3A. Sanger sequencing chromatogram of proband A1, with mutationc.2600C>T, p.A867V. Sequences aligned to virtual consensus with Mutation Surveyor (SoftGenetics, Inc.)

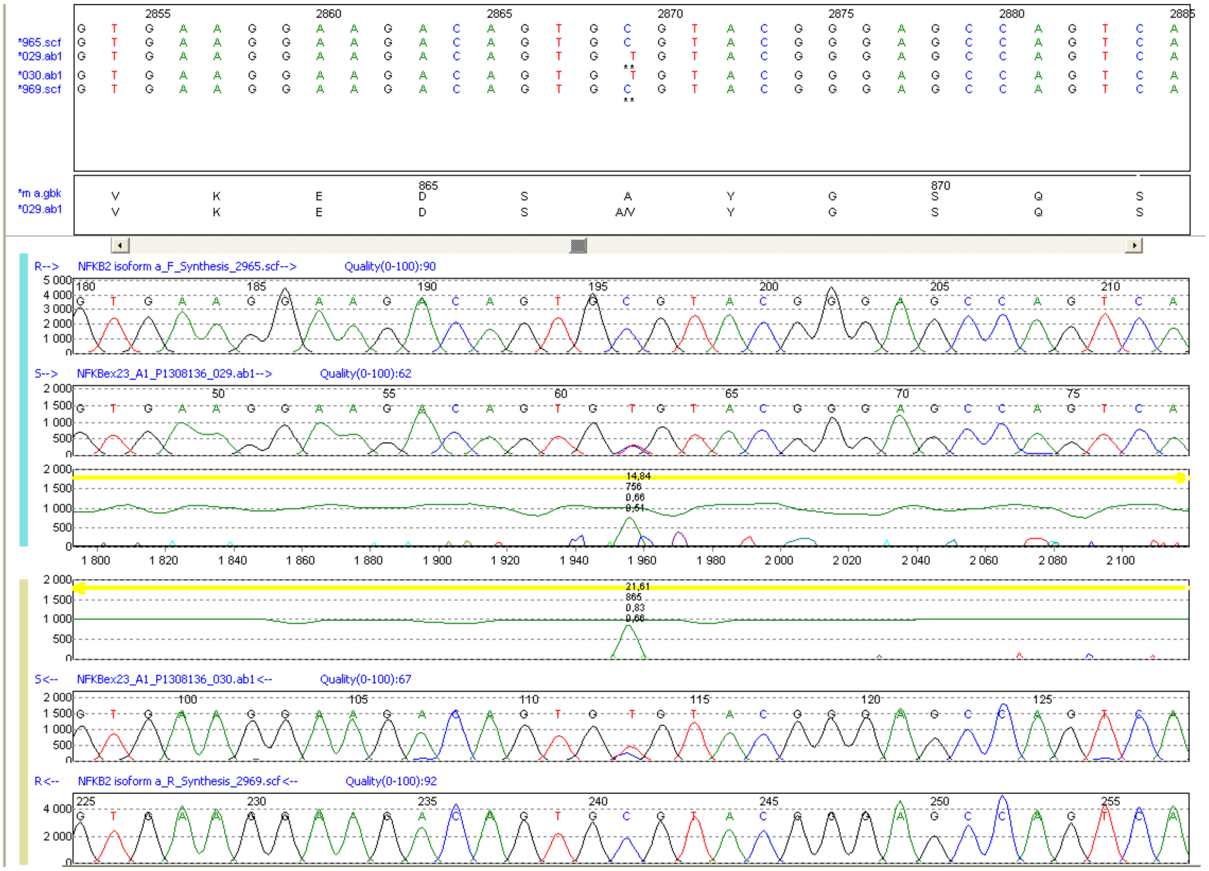

S3B. Sanger sequencing chromatogram of proband B1, with mutation c.2594A>G, p.D865G.

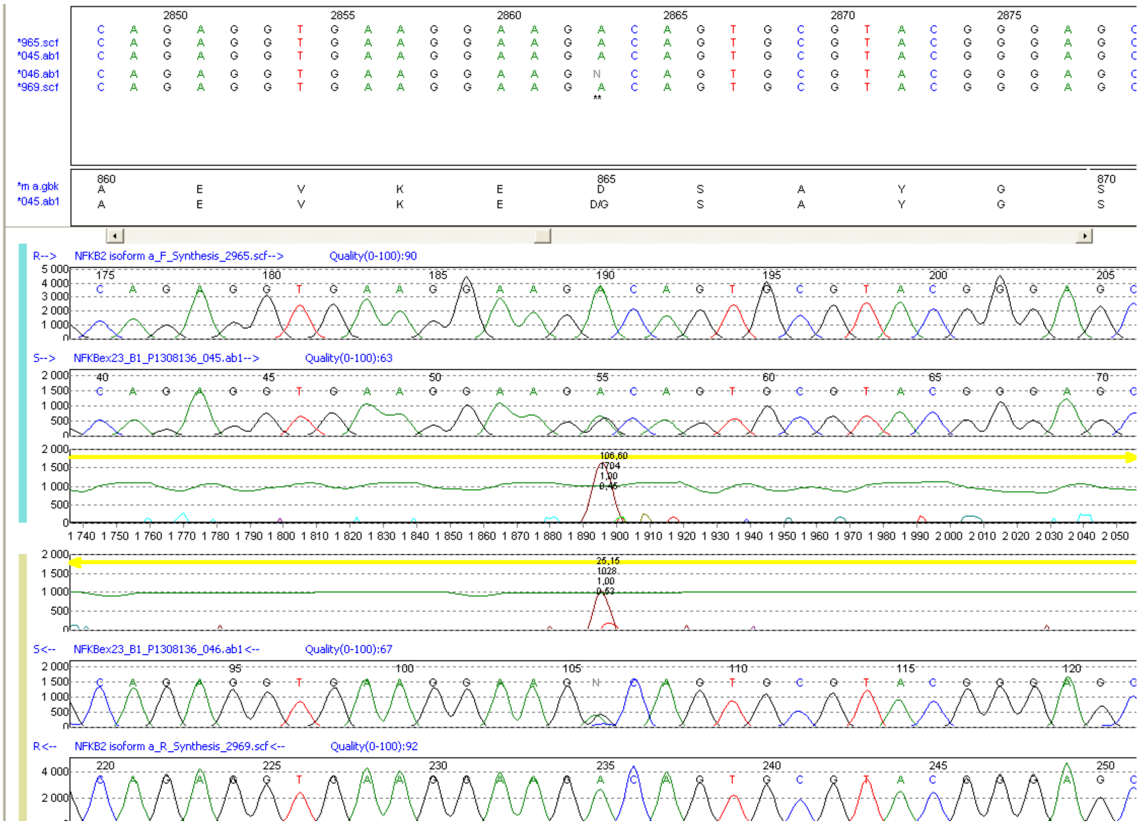

S3C. Sanger sequencing chromatogram of proband C1, with mutation c.2556_2563_delCCGAGACA, p. R853Afs*29.

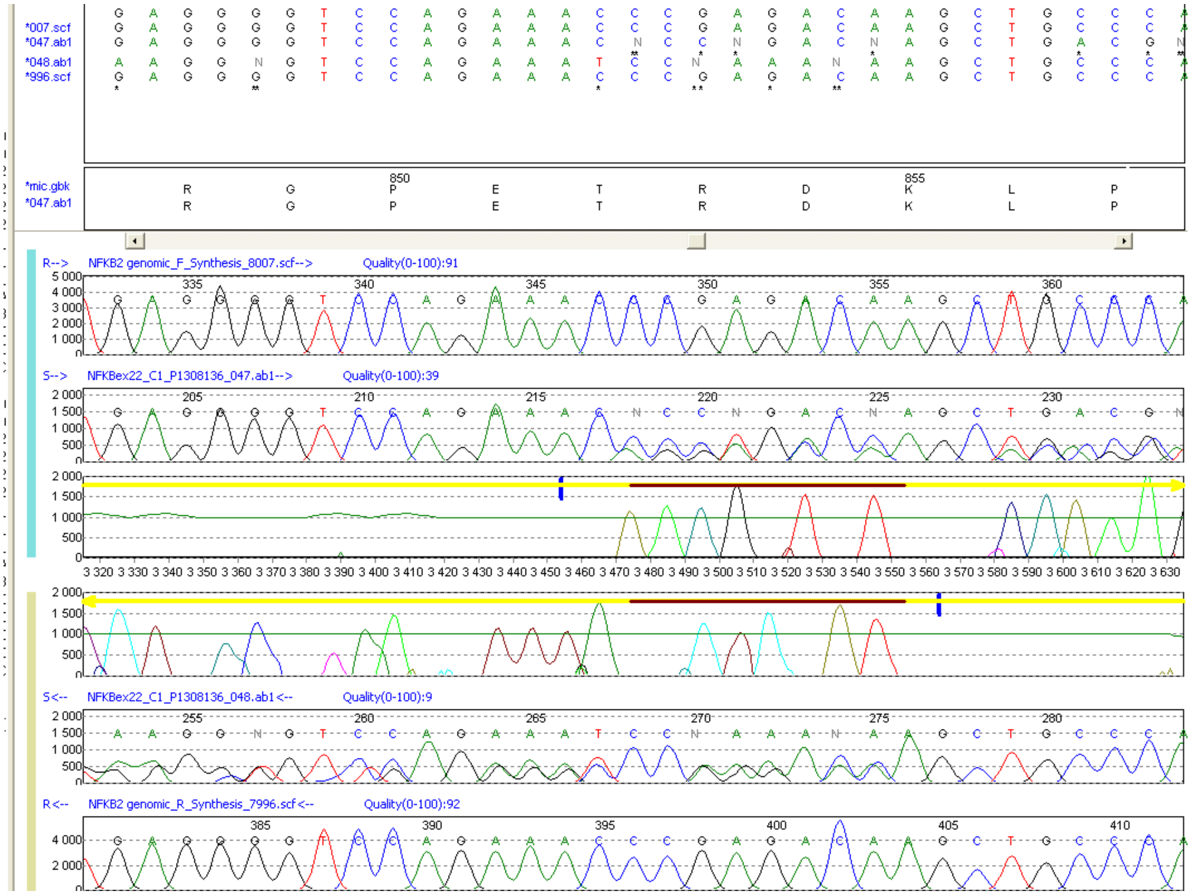

S3D. Deconvoluted frameshift mutation in proband C1, showing wild type and shifted mutation.

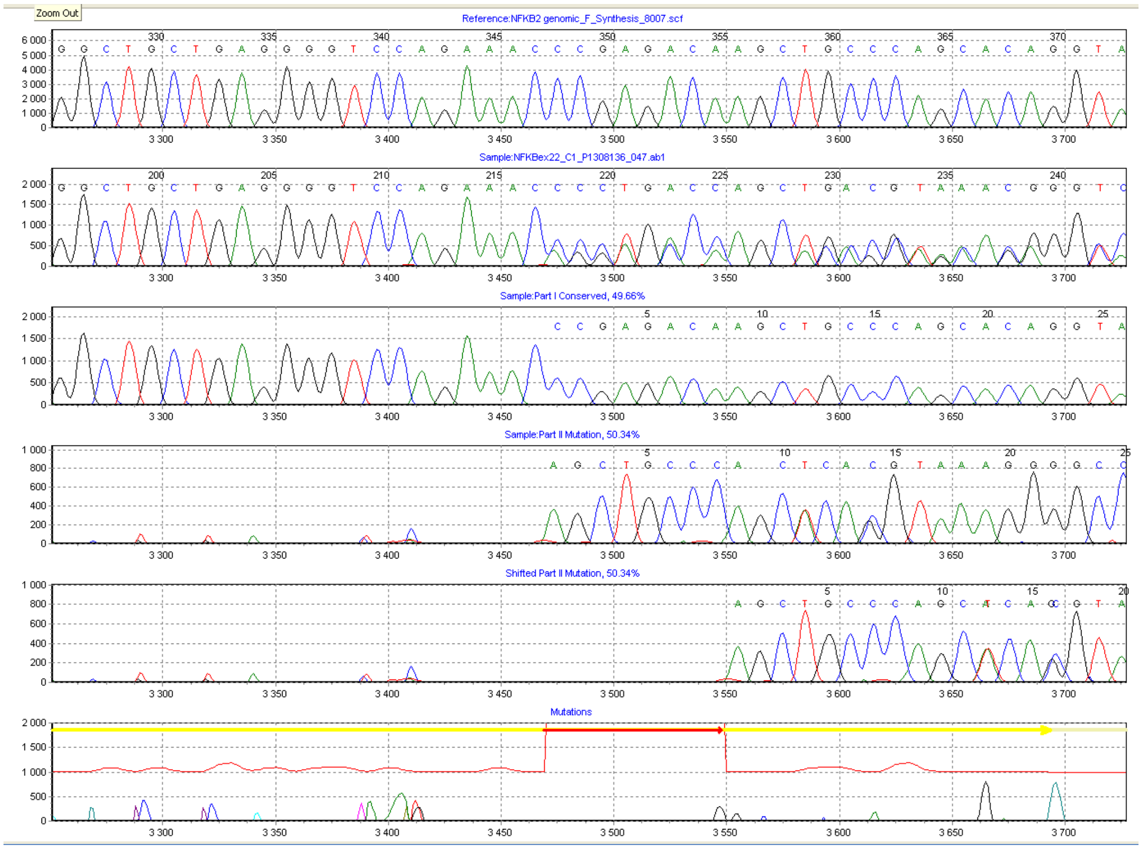

S3E. Sanger sequencing chromatogram of proband D1, with mutation c.2557C>T, p.R853X.

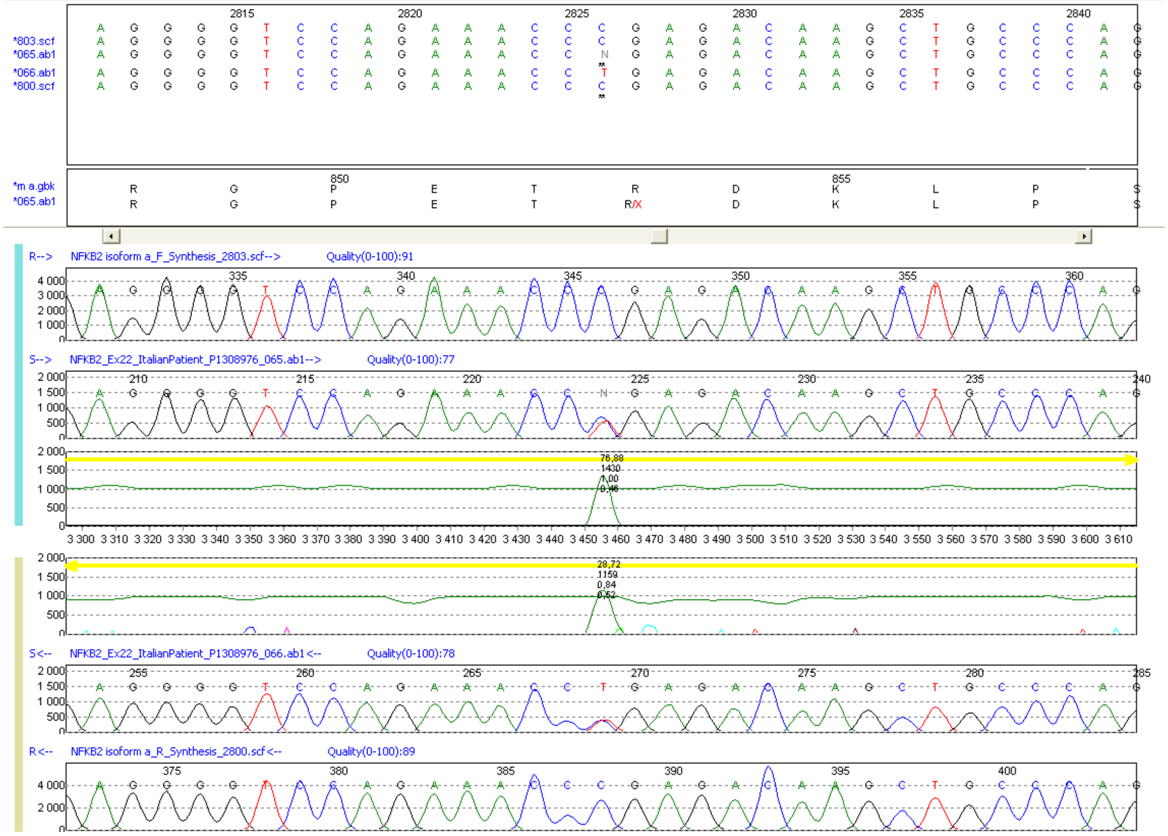

Figure S4. Histology of Lym1 mutant mouse pituitaries and adrenals. Staining for ACTH or Tpit as descrbed in Figure 2 legend, animals were age either 4 months (males shown, similar results for females, N=4 of each sex) or 8 months (females shown, males similar, N=3 for each sex).

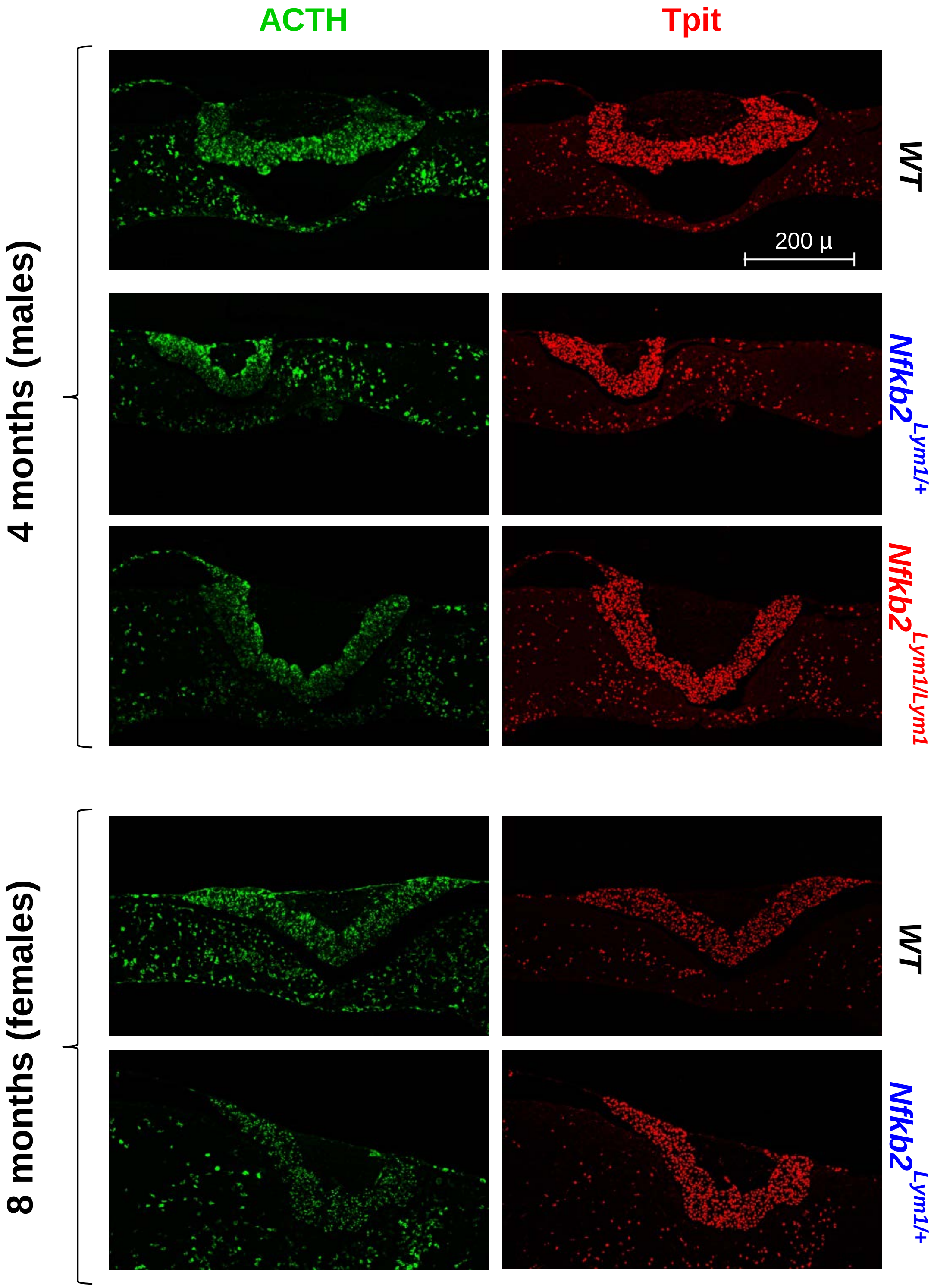

Figure S5. Nfkb1/2 expression in adult mouse pituitary cells. Bars correspond to the relative expression levels (+/- SD) of Nfkb1/2 determined by transcriptomic profiling (Affymetrix Gene 1.0 ST arrays) of FACS-sorted corticotropes (ACTH), melanotropes (MSH) and gonadotropes (LH).

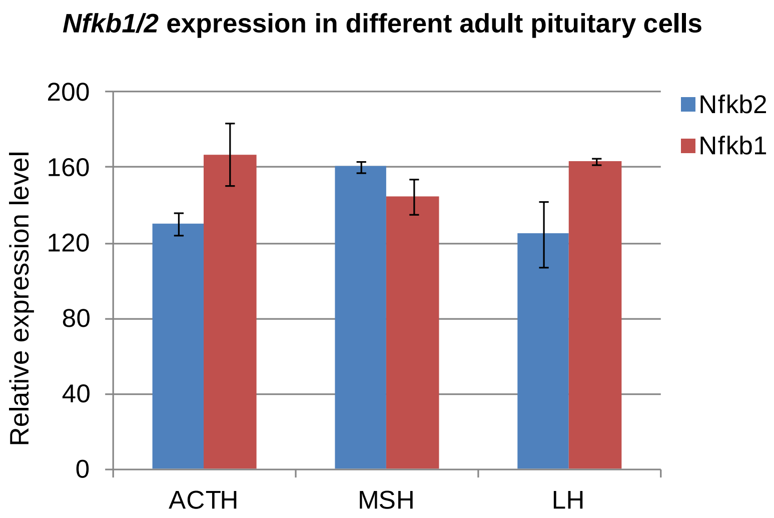

Supplement: Additional file 1: — Clinical description of the patients. [file 12881_2014_139_MOESM1_ESM.pdf]
